# Lectures in the Buffalo Medical College

**Published:** 1877-12

**Authors:** 


### Lectures in the Buffalo Medical College.

The regular lecture term in the Buffalo Medical College, commenced Nov. 7th, with a very unusually large class, the Hospital Amphitheatre for Clinics being for the first time much too small for their accommodation. The Medical Profession must be offering inducements to Doctors, which in Buffalo, is poorly appreciated. The clergy and even the women are trying to compete for its honors and rewards. As we look over the crowded Amphitheatre, we are force to repeat Abernethy's old prayer, new of course, every time it is repeated, "God have mercy on you all."

But we have never seen a more intelligent, attentive and attractive company of young men, whose earnest attention and manner speaks whole volumes for their future usefulness and success. The Medical profession of the future can be now seen in our Medical Colleges, and all true lovers of the profession, will take pride and pleasure in the character and standing of the young men composing the classes in our Medical Colleges.

The lectures upon Chemistry will be given by Dr. Charles A. Doremus, the talented son of the distinguished Professor, R. Ogden Doremus, of Bellevue Hospital Medical College, commencing Dec. 12th, 1877. This gentlemen brings the highest testimonials from the profession in New York, and a very instructive and pleasing course is anticipated.

———:o:———

### LACTOPEPTINE.

Pepsin is unquestionably a valuable remedy in some cases of indigestion, but does not seem to meet all the requirements of many dyspeptic cases. Lactopeptine is presented to the profession as meeting all the indications in cases of mal-nutrition and non-assimilation, composed according to the formula, of ptyalin, pepsin, pancreatine, hydrochloric and lactic, acids. It is claimed to be a combination of all the digestive agents. If we can prescribe chemically for disorder of the digestive function, such a combination would appear worthy of trial and experience has demonstrated its value in many cases. Dr. Merritt remarks, "the more my experience in its varied applicability extends, the more its beneficial effects appear."

